# Determinants of delayed diagnosis among pediatric cancer patients from Ayder Comprehensive Specialized Hospital, Mekelle, Northern Ethiopia

**DOI:** 10.1186/s12887-019-1848-1

**Published:** 2019-12-06

**Authors:** Alemseged Berhane, Tadele Hailu, Afework Mulugeta

**Affiliations:** 1 Department of Pediatrics and Child Health, Ayder Comprehensive Specialized Hospital, College of Health Sciences, Mekelle University, Mekelle, Ethiopia; 2 School of Public Health, Ayder Comprehensive Specialized Hospital, College of Health Sciences, Mekelle University, Mekelle, Ethiopia

**Keywords:** Facility based, Determinants, Paediatric cancer, Cross sectional, Ayder

## Abstract

**Background:**

Despite advances in the field of pediatric oncology, cancer remains a leading cause of death in children. The delays in cancer diagnosis may occur throughout the diagnostic pathway. Diagnosis of childhood cancer as early as possible is crucial to reduce mortality. The aim of this study was to identify determinants of delayed diagnosis among pediatric cancer patients in Ayder Comprehensive Specialized Hospital, Northern Ethiopia.

**Method:**

Facility based cross-sectional study was conducted among pediatric cancer patients aged less than 18 years. Data collection was done by interviewer-administered structured questionnaire from the volunteer primary caregiver from 1st September 2017 to 30th August 2018. The data was checked and cleaned by principal investigator on daily basis during data collection for completeness, consistencies, then coded, entered and analyzed using SPSS version 21 software. Diagnosis delay was considered significant when it is above the 3rd quartile. Binary logistic regression analysis was used to test associations between each of the determinant factors and the dependent variable. Variables with *P*-value < 0.25 during bivariate analysis were fed to the multivariate logistic regression model. Finally, variables with *P*-value < 0.05 were considered as determinants of delayed diagnosis.

**Results:**

From a total of 102 patients, 71(69.6%) had delayed diagnosis. Children older than 10 years of age were four (AOR = 4.01; 95%CI = 1.55–12, *P* < 0.001) times more likely to get delayed compared to under five children. Rural residence (AOR = 3.3; 95%CI = 1.24–10.24, *P* < 0.001), uneducated parents (AOR = 3.4; 95%CI = 1.91–13.25, *P* = 0.009), parents with monthly income less than 1000 ETB (AOR = 6.1; 95%CI = 1.76–7.23, *P* < 0.001), absence of health insurance (AOR = 2.4; 95%CI = 1.50–3.50, *P* = 0.02), visit to holy water (AOR = 3.4;95%CI = 1.6–7.2) and those who think cancer is incurable (AOR = 2.7;95%CI = 1.3–14,*P* = 0.004) were also likely to be delayed.

**Conclusion:**

Delayed diagnosis of childhood cancer was a major issue and most influenced by the child’s age, residency, family’s socioeconomic status, parental education, health insurance, use of holy water and caregivers perception on curability of cancer. Thus; every effort should be made to promote public and parental awareness of childhood cancer and promoting health insurance.

## Background

According to the 2016 estimates of the International Agency for Research on Cancer (IARC), 300,000 new cases of cancer in children less than 19 years appear in the world every year. Of these, 80% live in low and middle-income countries (https://www.acco.org/global-childhood-cancer-statistics). Cancer is an important cause of childhood mortality, with an estimated 80,000 cancer-related deaths per year worldwide with developing countries taking the largest share (https://www.acco.org/global-childhood-cancer-statistics). In LMIC advanced disease at presentation and delayed diagnosis of cancer in children is common, however there are limited studies regarding the determinants of delayed diagnosis [1–4].

The determinants of delayed diagnosis in developing countries varied across studies. Some of the factors associated with delay includes tumor type, health insurance and health care system. Studies from Western Kenya and Uganda found longest delay among Burkitt lymphoma [5], retinoblastoma and Hodgkin lymphoma from Nigeria [3, 6] and retinoblastoma patients from Kenya had longest median patient delay as well as physician delay [1]. South African study demonstrated considerable delay in diagnosing childhood cancer due mostly to a physician delay [7]. Absences of health insurance in Kenya was associated with delay and abandonment of treatment [1]. Dang-Tan et al. published a review of 23 studies that explore diagnosis delays in childhood cancer and revealed that the determinants of delay in developed countries also varied across studies [8].

Early diagnosis is fundamental for cancer management because it allows treatment of early stagedisease, which results in better prognoses [9]. The delays in cancer diagnosis may be related to the parents, the health personnel or the health system. Failure to recognize cancer symptoms may result in patient delay according to national survey from NHS patients [10]. Health care providers are expected to make the diagnosis of cancer as early as possible, but most cancer symptoms are vague and nonspecific making it difficult to detect early [11]. Knowing the different levels of delay is the first step to improve the care and treatment options given for the patients. However, most of the studies addressing delay were done in high-income countries [12–15].

In Ethiopia, there are approximately 6000 estimates of new cases of pediatric cancer each year, according to INCTR-USA hospital twinning initiative with Tikur Anbessa Specialized Hospital. Most children present late, with advanced disease [16].

There are no comparable studies available in Ethiopia about the determinants of diagnosis delay. But, we have observed that a lot of patients visit our center late and many days had elapsed even after visiting our center until the diagnosis of cancer. Once the diagnosis was made, treatment delay was not an issue; most patients started treatment with in few days of diagnosis. Hence, understanding the potential factors influencing the delayed diagnosis of cancer is needed to fill in the existing gap in knowledge and develop practical strategies to address the delay in cancer diagnosis among the pediatric population in Ethiopia.

## Method

### Study setting

Ayder comprehensive specialized hospital is a referral hospital found in Mekelle city, Ethiopia. It is the second largest hospital in the nation and has 500 inpatient beds in the four major departments and other specialty units. The hematology and oncology unit is under the Department of Pediatrics and Child Health with a separate wing in the ward with four trained nurses, one second-year pediatrics resident, and one hematologist and oncologist. It is highly organized unit with providing treatment to all types of pediatric cancer.

### Study population and design

A facility based cross-sectional study design was conducted. The subjects included all newly diagnosed pediatric cancer patients aged less than 18 years with a histological or cytological diagnosis of cancer admitted over the study period (i.e. from September 2017 to 30th August 2018) whose parents or care givers gave informed consent. Patient data was obtained within 1 week of diagnosis.

### Eligibility criteria

All cancer patients younger than 18 years of age were included. Patients who already were diagnosed with a malignancy in other facility and transferred for completion of treatment were excluded.

### Sample size and sampling method

All of the pediatric cancer patients (*n* = 102) who visited Ayder Comprehensive Specialized Hospital during the study period were included in the study. Consecutive sampling method was employed to select the study subjects from the Oncology ward of Ayder Comprehensive Specialized Hospital.

### Data collection

The questionnaire had been derived from extensive literature studies. Interviewer administered structured questionnaire was used to collect data from those patients caretaker’s after they are informed with the verbal consent form. The questionnaire was first developed in English and translated in to local languages (Tigrigna and Amharic), and then translated back in to English by the third person to check the consistency.

First, sociodemographic characteristics regarding the child and care giver was asked. To asses different levels of delay the patients were asked one question for patient delay, “What was the time taken from the onset of symptom to first contacting medical person?” Next, in the physician delay section, the patients were asked for details regarding delay between first contacting a medical person until diagnosis.. When appropriate, other family members who accompany the child or the child her/himself were interviewed to crosscheck the exact date.

These details included information on place of contact, whether a preliminary diagnosis was made. Patients were also asked about the possible reasons for such delay. Total diagnosis delay was also documented. Obviously, by summing the patient and physician delay. Further, patients file was used to cross check for available durations and to document the type of malignancy. Parameters regarding care givers knowledge and perception of cancer, reason of delay where assessed with open ended questions and then categorized.

### Data processing and analysis

The data were checked by the principal investigator on a daily basis during data collection for completeness, and consistencies. Data collected were then coded, entered, cleaned and analyzed using SPSS version 21. Descriptive analysis was used to describe the frequencies and percentages of the variables in the study. The strength of statistical association was measured by adjusted odds ratio and 95% confidence interval.

Binary logistic regression analysis was used to test associations between a single determinant and the dependent variable. Variables with *P*-value < 0.25 during bivariate analysis were included to the multivariate logistic regression model. Finally, variables with *P*-value < 0.05 were considered as potential determinants of delayed diagnosis among pediatric cancer patients.

Previous studies differ not only with definitions of delay but also with measurements of delay [9] and are thus, inconsistent. According to Dang-Tan et al. a review of 23 epidemiological studies that examined diagnosis delays in childhood cancer the levels of delay assessed varied across studies. Some researchers have focused on the time between a patient’s first symptom recognition to a diagnosis of cancer. This time period, called diagnosis delay has also been designated as lag time, wait time, pre-diagnosis symptomatic interval or time to diagnosis by different authors [8]. In the Mexican study time to diagnosis (which was labeled as “TD”) was defined as the time between the onset of symptoms and confirmation of diagnosis. By consensus, a diagnosis made 1 month after the onset of symptoms was considered a delayed time to diagnosis [17].

In our paper since the delay distribution is heavily skewed, the median would be a better choice of data presentation. Further, with lack of consistent definition we took the upper quartile (*3rd*) as significant diagnosis delay with consensus of all researchers who participated in this research.

### Definition

We defined the total diagnostic delay as the time interval from the detection of manifestations of disease to diagnosis of cancer. The patient delay was the time between the onset of signs and symptoms and the patient’s first visit to a health care, whereas the time elapsed from the first health care system contact to the diagnosis made up physician delay. Holy water refers to a freshwater in the form of stream or spring which runs close enough with the church’s compound. Otherwise, water must be blessed by a priest using the cross to be considered holy.

## Results

### Socio-demographic characteristics of study participants

A total of 102 participants were enrolled with newly diagnosed malignancy making response rate of 100%. About 70 (68%) of the study participants were males. Five patients were excluded from the outset as they were transferred from other set up for treatment completion. The age range of study participants ranged from 6 months to less than 18 years with mean and median of 10 years and 12 years, respectively**.** Similarly, 62(60.8%) of the participants were urban residents. Thirty-four (33.3%) of attendants were not educated**.** More than three-fourths of parents 78 (76.5%) were unemployed. Half of the parents have monthly income below 1000ETB and more than three fourth of them had no health insurance (Table 1).

**Table 1:** Socio-demographic characteristics of study participants and caregivers among pediatric cancer patients in Ayder Comprehensive Specialized Hospital, Tigray, Northern Ethiopia, 2018 (*n* = 102)

Variables /category	Delayed Diagnosis	
	Yes	No
Sex		
Male	49 (70%)	21 (30%)
Female	22 (68.7%)	10 (31.3%)
Age		
Less than 5 years	9 (40.9%)	13 (59.1%)
5–10 years	22 (78.6%)	6 (21.4%)
10 – ≤ 18 years	40 (76.9%)	12 (23.1%)
Residence		
Rural	34 (85%)	6 (15%)
Urban	37 (59.7%)	25 (40.3%)
Educational status of caregivers		
Educated	40 (58.8%)	28 (41.2%)
Uneducated	31 (91.2%)	3 (8.8%)
Care givers occupation		
Employed	18 (75%)	6 (25%)
Unemployed	53 (68%)	25 (32%)
Family Size		
Less than 5	40 (63.5%)	23 (36.5%)
Above 5	21 (53.9%)	18 (46.1%)
Monthly income		
Less than 1000	40 (78.4%)	11 (21.6%)
1000–3000	17 (77.3%)	5 (22.7%)
Above 3000	14 (48.3%)	15 (51.7%)
Health insurance		
Yes	12 (46.1%)	14 (53.9%)
No	59 (77.6%)	17 (22.4%)

### Health system literacy of caregivers

Patients’ first contact with a health-care provider were health centers for 41(40.2%) cases, general hospitals for 26(25.6%) cases, primary hospital for 20(19.8%) cases, private clinics for 10 (9.9%) cases and 5(4.9%) patients directly visited ACSH. The source of referral was mostly from general and primary hospitals (i.e. 46 cases). A significant number of patients 25(24.5%) visited holy water (Table 2).

**Table 2:** Health system literacy of caregivers among pediatric cancer patients in Ayder Comprehensive Specialized Hospital, Tigray, Northern Ethiopia, 2018 (*n* = 102)

Variables /category	Delayed diagnosis	
	Yes	No
First facility visited		
Health Center	30 (71.4%)	12 (28.6%)
Hospital	32 (72.7%)	12(%27.3)
Private Clinic	7 (63.6%)	4 (36.4%)
ACSH	2 (40%)	3 (60%)
Source for referral		
Health Center	27 (71.1%)	11 (28.9%)
Hospital	33 (76.7%)	10 (23.3%)
Private Clinic	11 (68.7%)	5 (31.3%)

### Levels of delay and reason for a delay

This study demonstrated that a delay in diagnosis among pediatric cancer patients was common in our settings, with much of the delay occurring prior to the first health encounter. The median patient delay was 50 days, the median physician delay was 32 days and the median diagnosis delay was 90 days. From the total of 102 patients, 71 (69.6%) experienced significant diagnosis delay i.e. > 170 day.

**Fig. 1:**
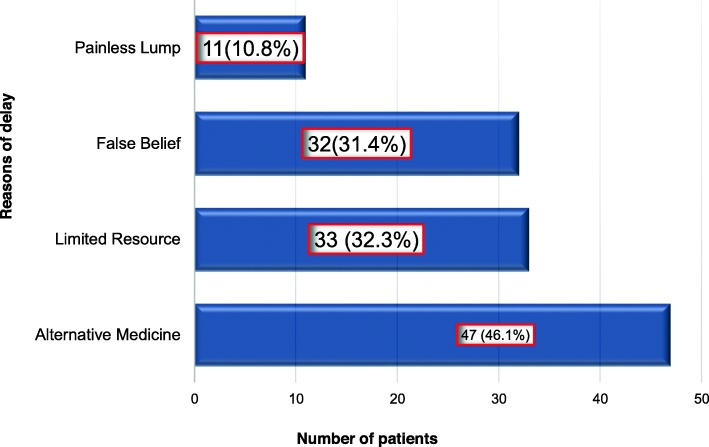
Reasons for delay reported by parents among pediatric cancer patients in Ayder Comprehensive Specialized Hospital, Tigray, Northern Ethiopia, 2018 (*n* = 102)

The main reasons for the delayed presentation were use of alternative medicine in 47 (46.1%) cases, limited resources in 33 (32.3%) of the cases, false belief regarding cancer in 32 (31.4%) cases, 11(10.8%) due to painless lump. Alternative medicines sought by parents included: holy water 25 (24.5%) cases, praying ceremonies 4 (3.9%) cases, visiting herbalist 12 (11.7%), use of over the counter drugs 3 (2.9%) and others (Fig. 1).

### Types of malignancy

**Fig. 2:**
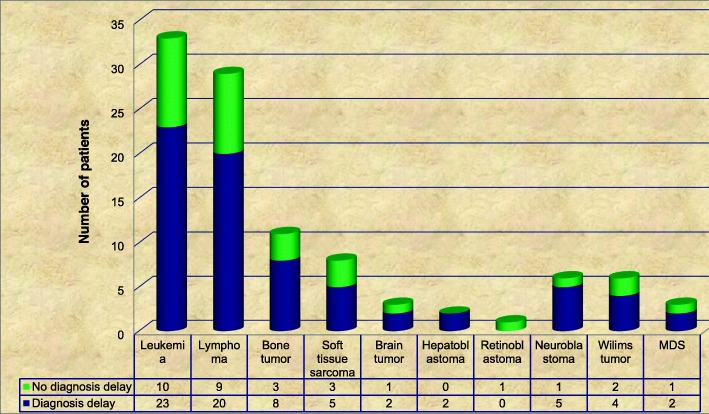
Types of malignancy among pediatric cancer patients in Ayder Comprehensive Specialized Hospital, Tigray, Northern Ethiopia, 2018 (*n* = 102)

The most common malignancy was leukemia 33 (32.3%) followed by lymphoma 29(28.4%) (Fig. 2).

### Referral diagnosis and evaluation at ACSH

**Fig. 3:**
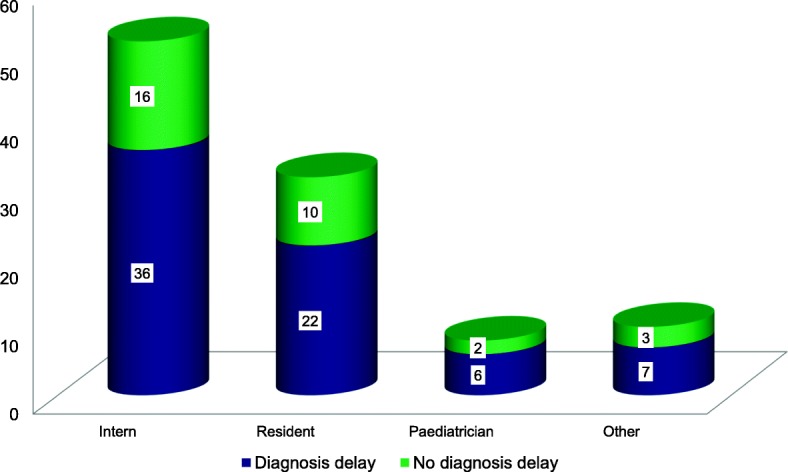
Initial evaluating health worker of cancer patients in Ayder Comprehensive Specialized Hospital, Tigray, Northern Ethiopia, 2018 (*n* = 102)

Before attending ACSH, cancer had already been clinically considered in 20 (21%) of the patients. Most patients 52(51%) were initially evaluated by interns, 32 (31%) evaluated by resident, 8(8%) evaluated by a pediatrician and the remaining 10 (9.8%) patients were evaluated out of pediatrics clinic. Diagnosis at ACSH was made with clinical evaluation and investigation modalities including bone marrow aspiration, bone marrow biopsy, FNAC, excisional biopsy and/or diagnostic imaging whichever is appropriate according to the clinically suspected malignancy (Fig. 3).

### Caregivers’ knowledge and perception of cancer

All of the caregivers have heard of cancer but 42(41.2%) of them think cancer is incurable. Fifty-seven of caregivers think that pediatric cancer has a worse outcome as compared to adults. (Table 3).

**Table 3:** Caregivers knowledge and perception of cancer among pediatric cancer patients in Ayder Comprehensive Specialized Hospital, Tigray, Northern Ethiopia from, 2018 (*n* = 102)

Variables /category	Delayed Diagn0sis	
	Yes	No
Curability of cancer		
Yes	30 (53.6%)	26 (46.4%)
No	40 (95.2%)	2 (4,8)
Uncertain	1 (25%)	3 (75%)
Pediatrics cancer outcome better than adult		
Yes	29 (67.4%)	14 (32.6%)
No	41 (71.9%)	16 (28.1%)
Uncertain	1 (50%)	1 (50%)

### Determinants of delayed diagnosis among pediatric cancer patients

The variables that exhibited significant association at *P* < 0.25 in bivariate analysis were simultaneously included to multivariate logistic regression. In multivariate analysis, the age of the child, the area of residence, caregivers’ level of education, family income, health insurance, visit of holy water and curability of cancer as reported by caregivers’ were found to be significantly associated with delayed diagnosis of cancer patients.

Children older than 10 years of age were four (AOR = 4.01; 95%CI = 1.55–12) times more likely to get delayed as compared to under five children. Those caregivers from the rural residence were 3 (AOR = 3.3; 95%CI = 1.24–10.24) times more likely to get diagnosed late relative to urban residents. Children’s of parents who never attended school were 3 (AOR = 3.4; 95%CI = 1.91–13.25) times more likely to get delayed as compared to those who attended primary school and above. Parents with a monthly income less than 1000 ETB were 6 times (AOR = 6.1; 95%CI 1.8–7.2) more likely to be delayed compared to those with monthly income of greater than 3000. In addition, those who have no health insurance were 2 times (AOR = 2.4; 95%CI 1.5–3.5) more likely to be diagnosed late as with compared to those who have health insurance. Similarly, visit of holy water (AOR = 3.4; 95%CI = 1.6–7.2) and those who think cancer is incurable were 3 times (AOR = 2.7; 95%CI = 1.3–14, *P* = 0.004) more likely to get delayed (Table 4).

**Table 4:** Bivariate and multivariable analysis on determinants of delayed diagnosis among pediatric cancer patients in Ayder Comprehensive Specialized Hospital, Tigray, Northern Ethiopia, 2018 (*n* = 102)

Variables and category	Delay Diagnosis		COR (95%CI)	AOR (95%CI)
	Yes	No		
Age of the child				
< 5 yrs	9 (12.7%)	13 (42%)	1	1
5 – 10 yrs	22 (31%)	6 (19.3%)	5.3 (1.53–18.3) *	2.19 (1.00–7.23)
> 10 yrs	40 (56.3%)	12 (38.7%)	4.81 (1.66–13.9) *	4.01 (1.55–12) **
Residency				
Rural	34 (47.9%)	6 (19.3%)	3.8 (1.40–10.46) *	3.3 (1.24–10.24) **
Urban	37 (52.1%)	25 (80.7%)	1	1
Educational status of caregiver				
Educated	40 (56.3%)	28 (90.3%)	1	1
Uneducated	31 (43.7%)	3 (9.7%)	7.23 (2.01–26) *	3.4 (1.91–13.25) *
Monthly family income				
< 1000 ETB	40 (56.3%)	11 (35.4%)	3.89 (1.45–10.46)*	6.1 (1.76–7.23) **
1000–3000 ETB	17 (24%)	5 (16.2)	3.64 (1.06–12.5) *	4.0 (0.2–39.9)
> 3000 ETB	14 (19.7%)	15 (48.4)	1	1
Health insurance				
Yes	12 (16.9%)	14 (45.2%)	1	1
No	59 (83.1%)	17 (54.8%)	4.04 (1.58–10.37) *	2.4 (1.50–3.50) *
Is cancer curable				
Yes	32 (53.6%)	24 (46.4%)	1	1
No	38 (95.2%)	4 (4.8%)	2.8 (2.23–22.68) **	2.7 (1.3–14.0) *

NB. *P < 0.05, ** *p* < 0.001

## Discussion

The aim of this study was to identify determinants of delayed diagnosis of pediatric cancer patients at Ayder Comprehensive Specialized Hospital. Earlier presentation leads to early diagnosis of cancer which is a fundamental goal in oncology because it allows an opportunity for timely treatment. Consequently, the prognosis may improve, and a cure can be attained with minimal side effects. In the current study, the age of the child, the area of residence, parent’s level of education, family income and health insurance were all found to be statistically significant determinants of delayed diagnosis of pediatric cancer patients.

Delay in diagnosis among pediatric cancer patients was common in our settings, with much of the delay occurring prior to the first health encounter. About 71 (69.6%) had significant total diag nosis delay i.e. > 170 days. The median patient delay was 50 days, the median physician delay was 32 days and the median diagnosis delay (i.e. patient delay + physician) was 90 days. The Kenyan and Nigerian total delays were 102 and 110 days respectively which was longer than this study [1, 2]. This could be explained by the inclusion of treatment delay in the studies from Kenya and Nigeria. Studies from Egypt and South-Africa reported similar findings with a median total delay of 47 and 34 days, respectively. The short diagnosis delay reported from South-Africa could be a reflection of the superior infrastructure which was similar to the delays found in high-income countries, such as Canada [4, 7, 13]. The main reasons for the delayed presentation were use of alternative medicine in 47(46.1%). Alternative medicines sought by parents included: holy water 25 (24.5%) cases, praying ceremonies 4 (3.9%) cases, visiting herbalist 12 (11.7%), use of over the counter drugs 3 (2.9%) and others. A study done in Kenya reported that more than half of patents have used alternative medicine prior to admission [1]. The use of holly water was unique in our setting which was sought by significant number of patients (i.e. 25). There are many mentions of holy water in Ethiopian church literature [17]. Despite holy water is considered a cure to a variety of illness in Ethiopia, literature is scarce. This could be due to the highly spiritual community [18].

Age of the children was a significant predictor of delayed diagnosis. Fajardo-Gutierrez et al. found that the risk of increased delay for children between the ages of 10 years and 14 years is 1.8 times that of infants younger than 1 year of age (OR = 1.8(95% CI, 1.4–2.3) [17]. Another Kenyan study showed that the longest total delay was for the age group 6–10 years though it was not significantly longer when compared to the other age groups [1]. It was also reported that diagnosis delay was shortest for children aged 0–2 years despite no significant differences in histopathology, grade or location of tumors [18]. Those younger children had the shortest delay which could be because the common tumors in this age group are aggressive and may lead to a faster presentation and earlier diagnosis.

This study did not find a significant difference in the diagnosis delays between the two genders which is consistent with many studies. The sex of the child didn’t influence diagnosis delay in different studies [3, 4, 7, 15]. This may imply that there are no differences in health-seeking behavior regardless of the gender of the child.

The fact that children are usually under the care of their parents, parental characteristics and behaviors are also important factors in recognizing symptoms and signs of cancer. Thus, socioeconomic status and parental education were also identified as determinants of delayed diagnosis in this study. Parents who never attended school were more likely to be diagnosed late as compared to those who attended primary school and above school. In the Argentinean study, patients whose parents had an elementary education or lower had a greater risk of longer patient delay [18]. This was also observed in Mexico, where children whose parents had the lowest level of education had longer delays in diagnosis than children with parents with the highest level of education (OR = 1.4 for fathers and 1.5 for mothers) [17]. The rural residency was also associated with a significant delay in diagnosis which was consistent with Nigerian study where patients with longer travel distance and rural residence was associated with a delayed presentation [2]. This could be due to long travel distance and lack of transportations.

Parents with monthly income less than 1000 ETB were around 6 times more delayed in this study. This was consistent with a study done in Egypt where lower socioeconomic status and delayed diagnosis showed statistically significant correlations (*P* < 0.001) [4].

Having health insurance resulted in significantly shorter diagnosis delay compared to those without. This is consistent with a study done in Kenya where having insurance reduced the risk of abandonment [19]. Another study done in Kenya showed that health-insurance at diagnosis resulted in a significantly less patient delay (*P* = 0.049) [1].

Pediatric cancer patients who used holy water prior to first health encounter were 3 times more likely to get delayed and those who think cancer is incurable were 3 times delayed than those who think that cancer is curable. The use of holly water was unique in our setting which was sought by significant number of patients (i.e. 25). There are many mentions of holy water in Ethiopian church literature [19]. Despite holy water is considered a cure to a variety of illness in Ethiopia, literature is scarce. This could be due to the highly spiritual community.. Other factors which were found to be determinants in different studies including the type of cancer, parental occupation, and type of referring facility were not associated with delayed diagnosis in this study [1, 14, 20]. Therefore, some associated factors that were found to be determinants of delayed diagnosis in one study may not necessarily be determinants in another study supporting the argument that possible determinants of delay differ across the geographical location because of socioeconomic differences, beliefs and health-seeking habits of the specific community and other factors.

This study had certain limitations, including its relatively small sample size and recall bias. Recall bias was an issue when asking about the beginning of symptoms, but we attempted to achieve accuracy by crosschecking with other family members, reviewing dates of referral letters and initial written suspected diagnoses.

## Conclusion

Delay in diagnosis among pediatric cancer patients was common in our settings, with much of the delay occurring prior to the first health encounter. The median patient delay was 50 days, the median physician delay was 32 days and the median diagnosis delay was 90 days. About 71 patients had significant diagnosis delay. These findings are more or less similar to other studies in LMIC. Delayed diagnosis of childhood cancer was most influenced by the child’s age, residence, family’s socioeconomic status, and parental education, absence of health insurance, visit of holy water and caregiver’s perception on curability of cancer.

Much of the delay occurred prior to the first health encounter. Thus, every effort should be made to promote public and parental awareness of childhood cancer and promoting health insurance to those with low socioeconomic status. We also recommend that training on childhood cancer be incorporated into the curricula of medical training institutions.

## Data Availability

The dataset used and/or analysed during the current study is available from corresponding author on reasonable request.

## Abbreviations

ACSH: Ayder Comprehensive Specialized Hospital
CT: Computed Tomography
ETB: Ethiopian Birr
IARC: International Agency for Research on Cancer
INCTR: International Network for cancer treatment-United States of America
LMIC: Low and Middle Income Countries
MDS: Myelodysplastic Syndromes
SPSS: Statistical Package for Social Sciences
TB: Tuberculosis
